# Professional Support Through a Tailor-Made Mobile App to Reduce Stress and Depressive Symptoms Among Family Caregivers of People With Dementia: Mixed Methods Pilot Study

**DOI:** 10.2196/75113

**Published:** 2025-09-30

**Authors:** Aber Sharon Kagwa, Jessica Longhini, Muhammed Nazmul Islam, Sofia Vikström, Åsa Dorell, Hanne Konradsen, Zarina Nahar Kabir

**Affiliations:** 1 Division of Nursing, Department of Neurobiology, Care Sciences and Society, Karolinska Institute, Alfred Nobels Allé 23, Huddinge, Stockholm, 141 83, Sweden, 46 702257856; 2 Laboratory of Studies and Evidence-based Nursing, Department of Cardiac, Thoracic, Vascular Sciences and Public Health, University of Padova, Padua, Italy; 3 Department of Research and Evaluation, Sajida Foundation, Dhaka, Bangladesh; 4 Division of Occupational Therapy, Department of Neurobiology, Care Sciences and Society, Karolinska Institutet, Huddinge, Stockholm, Sweden; 5 Department of Geriatrics, Hvidovre Hospital, Hvidovre, Denmark; 6 Steno Diabetes Center, Copenhagen, Denmark

**Keywords:** caregiver stress, dementia, dementia care, depressive symptoms, eHealth, mHealth, mobile app, social care professional, support, mobile health, informal caregiver

## Abstract

**Background:**

Providing informal care to people with dementia living at home can be challenging and may cause caregiver stress and depression. Interventions delivered through mobile apps provide innovative solutions for community-based social care professionals to address the increasing support needs of family caregivers (FCs) of people with dementia.

**Objective:**

This study aimed to examine, among FCs of people with dementia living at home, (1) the potential association between professional support provided through a mobile app and caregiver stress and depressive symptoms, (2) types of support provided through chat interactions between FCs and social care professionals, and (3) how support provided through a mobile app relates to changes in caregiver stress and depressive symptoms.

**Methods:**

A mixed methods pilot study integrated quantitative pre- and postintervention data with qualitative logged chat data. FCs of people with dementia living at home (n=35) were recruited to test a tailor-made mobile app over 8 weeks. The primary and secondary outcome measures were caregiver stress and depressive symptoms, respectively. Descriptive statistics were used to summarize sociodemographic factors; inferential statistics were used to analyze mean differences in outcomes pre- and postintervention. FCs were divided into 3 groups based on changes in caregiver stress scores between pre- and postintervention. Generalized linear model analyses determined the association between participation in the intervention and caregiver stress and depressive symptoms, adjusting for age, gender, and relationship to the person with dementia. Logged chat data were analyzed using summative content analysis to identify types of support provided and received. Changes in caregiver stress were integrated with chat data to determine patterns in types of support received.

**Results:**

The mean age of FCs was 69.4 (SD 11.9) years, with most being women (28/35, 80%), partners (24/35, 68.6%), and living with the person with dementia (26/35, 74%). The mean score of caregiver stress was marginally higher postintervention (24.1, SD 9.3) than preintervention (23.9, SD 9.2), whereas the mean score of depressive symptoms decreased from pre- (6.5, SD 5.1) to postintervention (6.2, SD 5.2). These differences were not statistically significant. Regression analyses showed that participation in the intervention was not statistically significantly associated with caregiver stress (β=0.171, α=.05; *P*=.86) or depressive symptoms (β=−0.293, α=.05; *P*=.75) after adjusting for age, gender, and relationship to the person with dementia. However, mixed methods analysis at the subgroup level suggested that frequent tailored support by social care professionals delivered through a mobile app may reduce caregiver stress among FCs of people with dementia living at home.

**Conclusions:**

The study highlights the importance of providing frequent and individualized support to meet the needs of FCs of people with dementia. Findings from this study may help community-based social care providers plan and organize digital support content provided to FCs of people with dementia living at home.

## Introduction

### Dementia

Dementia is a progressive condition with no current cure [1]. The condition is considered a global public health challenge and is the seventh leading cause of death worldwide [2]. It is characterized by impairments such as cognitive, functional, and behavioral [1] affecting the individual’s memory, behavior, and daily functioning [2]. In 2020, the number of people living with dementia worldwide was 55 million, with a projection of 139 million in 2050. In Sweden, 150,000 people lived with dementia in 2019, with a projection of 250,000 by 2050 [3].

### Being a Family Caregiver to a Person With Dementia

Most of the care provided to people with dementia in communities is provided through informal care by unpaid family caregivers (FCs) such as family members and friends [4]. Therefore, FCs play an integral part in the health care system, providing everyday care that is complex, challenging, and prolonged [1]. In 2019, the World Health Organization (WHO) estimated that FCs, on average, provided care and supervision to a person with dementia for 8 hours per day [5]. Most FCs of people with dementia in Sweden are older partners (including spouses and cohabiting partners) and women [6], and a recent study reported that about half of spouses of people with dementia provide care for ≥30 hours per week on average [7].

Positive aspects of informal caregiving include feeling satisfaction and pride in contributing to the quality of life of the person with dementia [8]. However, FCs often face challenges managing the person with dementia’s complex and challenging symptoms of dementia such as restlessness, agitation, and physiological distress [9]. The increased dependency of the person with dementia as the condition deteriorates poses another challenge for FCs, often leaving them overwhelmed [8]. The added responsibilities of FCs occur gradually and tend to increase over time as the condition progresses. This may lead to constraints in their daily lives, such as having to leave work [10]. The challenge of informal caregiving may lead to caregiver stress, which is multifaceted and related to dimensions such as psychological, physical, and social burden due to factors including limited time resources, required vigilance, and supervision [11]. The challenges of informal caregiving can also lead to caregiver depression. The risk factors of depression among FCs of people with dementia include being a woman, being a spouse, and cohabitating with the person with dementia. Caregiver depression among FCs of people with dementia is also related to functional decline and mortality [12].

### Support for FCs to People With Dementia in Sweden

The formal support provided to FCs in Sweden is most commonly indirect, in that the support is aimed toward the care recipient (eg, home care). Direct support to FCs is often provided by social care professionals in the municipalities. The educational backgrounds of social care professionals vary and include nurses, social workers, and nurse assistants. Social care professionals have an essential role in the development and coordination of support for FCs, including providing counseling through individual and group support and through education (eg, coping strategies) [13,14]. This type of support provided to FCs in Sweden [15] is often divided into three types of resources: (1) instrumental (ie, material aids and help with daily tasks), (2) informational (ie, relevant information, education, and provision of knowledge), and (3) emotional (ie, empathy, care, and validation) [15].

The National Board of Health and Welfare, through the Social Services Act (SOSFS; 2001:453), obligates municipalities to provide support to informal caregivers [16]. Despite this, most FCs are reported to be unaware of the available support services and who to turn to for support [6]. A study including FCs, people with dementia, and professionals conducted in eight European countries, including Sweden, explored the barriers and facilitators to accessing and using formal dementia care. The study found that a lack of knowledge about dementia and available services among FCs of people with dementia contributed to them not seeking needed support [17]. FCs of people with dementia reported retrospectively that they had waited too long to make use of professional support [17]. In line with this, research shows that FCs often delay seeking formal support services and neglect their personal needs [18] until experiencing a crisis [17,18], such as a sudden inability of the FCs to provide care to the person with dementia or behavioral symptoms of the person with dementia [17]. External support is also often sought when FCs no longer perceive they can function effectively due to increased responsibilities [10]. The competencies of social care professionals, including specific knowledge of dementia and having a key contact person, are important for access to formal care services [17]. In addition, a recent systematic review [19] aimed at identifying the needs of FCs of people with dementia living at home found that receiving tailor-made information to meet one’s own needs as FCs was important [19].

### Mobile Health–Based Support

The increasing need to support FCs of people with dementia has brought forth the development of innovative interventions delivered through mobile health (mHealth) technology, such as mobile app [20]. mHealth is defined by the WHO [21] as a medical and public health practice supported by mobile devices, such as mobile phones and other wireless devices. The benefits of mHealth technologies are that they are accessible and therefore reported to be easily incorporated into the daily lives of FCs of people with dementia when time permits [22]. The accessibility of mHealth technologies also increases the potential of reaching people who live long distances from health care services and those who are unable to meet in person [23]. Another benefit of mHealth technologies is their potential to provide cost-effective support that is personalized and needs-based by facilitating the connection between the health care provider and the health care recipient in real time [23]. However, a recent meta-analysis found that there was a limited number of interventions using mobile apps targeting FCs of people with dementia living at home [24]. There are also limited available apps that are directed toward the personal needs of FCs, such as stress management [25]. Furthermore, there are limited studies that assess the effectiveness of interventions through mobile apps in terms of health-related outcomes such as caregiver stress and depression of FCs of people with dementia living at home [24].

### The Intervention Tool STAV (STöd till AnhörigVårdare)

Considering the aforementioned gaps and the potential use of mHealth technologies in bridging these needs, we developed a tailor-made mobile app named STAV (STöd till AnhörigVårdare: support to FCs) with Karolinska Institutet eHealth Core Facility and byBrick Elevate [26]. The mobile app STAV consisted of (1) a chat feature that enabled FCs and social care professionals to interact, (2) a digital diary that enabled FCs to note any stressful or upsetting events such as behavioral changes in the person with dementia, (3) a mindfulness feature offering access to 3 guided sessions, each averaging 5 minutes in length, (4) a contact feature for registering relevant contact information, and (5) a web links feature with a curated collection of updated and relevant information regarding caregiving and dementia. The app was available on both iOS (Apple Inc) and Android (Google) digital platforms [26]. STAV was developed based on ideas and insights from user perspectives following individual interviews with FCs of people with dementia living at home and a focus group interview with community-based social care professionals specialized in dementia care [27]. The benefits of receiving support through the app for FCs have previously been reported, including offering a foundation for inner calm and relevant information in one place [28]. To gain a more comprehensive understanding of the potential of professional support delivered through the mobile app STAV, a mixed methods research design was used [29]. This approach enabled us to gain insights into how changes in the quantitative outcomes were related to the type of support provided by social care professionals as recorded in chat conversations, an approach that has rarely been used [30]. This study aimed to examine, among FCs of people with dementia living at home, (1) the potential association between professional support provided through STAV and caregiver stress and depressive symptoms, (2) the types of support provided through chat interaction between FCs and social care professionals, and (3) how provision of support through a mobile app relates to changes in caregiver stress and depressive symptoms.

## Methods

### Study Design

An explanatory sequential mixed methods design was used in this study. This method is useful when applying qualitative data to explain quantitative results [29]. The pilot study adhered to the Good Reporting of A Mixed Methods Study (GRAMMS) checklist [31].

### Setting and Participants

The study was conducted among FCs of people with dementia living at home in municipalities located in three regions of Sweden: Stockholm County, Västerbotten County, and Skåne County. The social care professionals who provided support through the chat feature of STAV were purposively sampled and worked in a municipality in Sweden, providing community-based support to FCs of people with dementia living at home. The social care professionals who participated in the study consisted of registered nurses specialized in dementia care, dementia coordinators, social workers, social aid officers, and nurse assistants specialized in dementia care, and worked in the municipalities or senior day care centers for people with dementia. The FCs of people with dementia were purposively recruited through the networks of social care professionals. Other channels of recruitment were local health care centers, websites, newsletters, and social media channels of the municipalities, dementia-related associations, and FCs’ networks. The eligibility criteria for the FCs included being an adult aged older than 18 years, who provided care for a person with dementia living at home for a minimum of 6 months, could read and write Swedish, possessed a smartphone or tablet or both, and had access to the internet at their own expense [26]. STAV was available on both iOS and Android devices. The participants received a brief introduction to STAV and, when needed, technical support by a member of the research team either via the telephone, in person, or both. Forty-six FCs were recruited for the intervention, all of whom completed the intervention, including the preintervention assessments. Of the 46 FCs, 11 (23.9%) FCs were lost to follow-up. Therefore, a total of 35 FCs were included in this study. This sample size is considered sufficient for a pilot study [32,33].

### The Intervention

The 8-week intervention in terms of professional support was delivered by community-based social care professionals through the chat feature of STAV. The support was delivered through synchronous and asynchronous chat conversations tailored to the individual needs of the FCs [26]. The other support features of STAV—the contact, the diary, web links, and mindfulness feature—were also available for the FCs to use at any time. Before the study commenced, social care professionals received an information package containing details about the intervention and the intervention tool STAV [26]. The social care professionals also participated in an introductory meeting, either in person or online, with members of the research team, to become familiar with the project. The intervention was implemented within the participants’ existing social care context and was tailored and delivered based on the FCs’ individual needs. As such, no additional training or retraining on the support content was provided beyond the initial introduction. However, regular contact was maintained between a member of the research team and the social care professionals throughout the intervention period. FCs were also given a brief introduction to the intervention tool STAV. In addition, the backend server where the logged chat was stored was monitored regularly. Members of the research team were available to provide technical support to both the social care professionals and the FCs when needed.

### Data Collection

The 8-week intervention had 2 data collection time points for the quantitative element (preintervention and immediately after the intervention). Data were collected through self‐administered questionnaires. Preintervention data were collected from the FCs through the mobile app STAV after obtaining informed consent and after they had downloaded the app. Follow-up data were collected through postal questionnaires.

Data for the qualitative element were collected using logged chat data based on chat conversations between the FCs and the social care professionals during the 8-week intervention. The logged chat data were stored in a back-end server of STAV and hosted by Karolinska Institutet.

### The Data

#### Quantitative Background Variables

The background variables included sociodemographic characteristics such as age (in years), sex (female or male), relationship to the person with dementia (partner [including spouse and cohabitating partner] or others [ie, adult children]), as well as living arrangements of the person with dementia (living with a family member or living alone).

#### Quantitative Outcome Variables

##### Caregiver Stress

Caregiver stress, the primary outcome, was measured using the validated Swedish version of the 12-item Zarit Burden Interview (ZBI-12) Short form [34], which captures the subjective feelings of burden derived from the caregiving role. The ZBI includes 12 items that measure the multidimensional aspects of burden, such as interpersonal relationships, FCs’ health, and emotional well-being. The tool includes a five-point response scale with the options (0=never; 1=rarely; 2=sometimes; 3=frequently; 4=or nearly always) [35]. A summary score ranging from 0 to 48 was calculated, where higher scores indicate higher burden (0‐10=no to mild burden, 10‐20=mild to moderate burden, >20=high burden) [36].

##### Depressive Symptom

The Swedish version of the Patient Health Questionnaire (PHQ-9) was used to measure the secondary outcome of depressive symptoms [37]. The PHQ-9 consists of 9 criteria on which the diagnosis of depressive disorders is based in the *DSM-IV* (*Diagnostic and Statistical Manual of Mental Disorders, Fourth Edition*) [38]. The validated tool includes a 4-point response scale ranging between 0=not at all, 1=several days, 2=more than half the days, and 3=nearly every day. A summary score ranging from 0 to 27 was calculated, with higher scores indicating greater symptom severity: 0-4=none, 5‐9=mild, 10‐14=moderate, 15‐19=moderately severe, and 20‐27=severe [37].

##### Qualitative Logged Chat Data

The chat conversations through the app between the FCs and the social care professionals were extracted from the back end of STAV into a Microsoft Excel sheet. The chat entries were matched using participants’ identification numbers for both the FCs and the social care professionals. The data consisted of text and, in some cases, emojis. The length of the text in each chat entry ranged from a single word or emoji to 102 words for the FCs and 103 words for the social care professionals.

### Data Analysis

#### Quantitative Analysis

There were no missing values for the preintervention assessments. For the postassessments, there were missing values for caregiver stress (3/35, 8.6%) and depressive symptoms (2/35, 5.7%). The missing values were replaced using the individual mean imputation method, in which the imputed value was based on the calculated mean of the participants’ complete responses to other questions for the specific instrument [39]. Descriptive statistics using frequencies, means, and SDs were calculated to describe the study sample, background characteristics, and outcome variables. A paired sample *t* test was used to compare the means of caregiver stress (ZBI-12) and depressive symptoms (PHQ-9) between pre- and post-intervention.

The Shapiro-Wilk test was not significant for caregiver stress at pre- and post-intervention, suggesting that the pairwise differences were normally distributed. The data were not normally distributed for depressive symptoms at pre- and post-intervention; therefore, a related-samples nonparametric test, the Wilcoxon signed-rank test, was conducted. The data were analyzed using SPSS (version 29; IBM Corp) and Stata (version 18.0; StataCorp).

#### Linear Regression

Thereafter, generalized linear multivariable regression analyses were conducted after checking for multicollinearity. These analyses examined the associations of the outcome variables, caregiver stress and depressive symptoms, with participation in the intervention (pre- vs post-intervention), controlling for age, sex, and relationship to the person with dementia.

#### Qualitative Analysis

The data were analyzed using summative content analysis [40]. In the initial step, the chat entries were counted by each participant. Thereafter, the logged chat data were read through repeatedly to familiarize with the data. On reading through the chat contents, patterns related to support were observed. The observed patterns of support were then analyzed against the types of support identified in a 2019 national FC mapping [15], namely instrumental, emotional, and informational support. In the analysis process, some of the chat messages were merged or split to accurately reflect the type of support when analyzing the data. To increase trustworthiness, the data were coded according to the type of support and described based on what each type of support included, following discussion and consensus among coauthors [41]. Trustworthiness was further enhanced by using Microsoft Excel to manage and track the coding process. This included detailed documentation of how chat message entries were counted and how different types of support were identified within the logged chat data. This structured approach contributed to both dependability and confirmability by providing a transparent and organized method for data handling, thereby reinforcing the credibility of the findings [41].

#### Mixed Methods Analysis

The quantitative and qualitative data were integrated by merging the chat log data with the quantitative primary outcome, caregiver stress, through the caregiver’s identification numbers. First, the FCs were divided into 3 groups based on the change in caregiver stress scores from pre- to post-intervention. Group 1 included those with decreased scores in caregiver stress, group 2 showed no change, and group 3 included those with increased scores. Each group was further examined in terms of change in depressive symptom scores and the content of the type of support as identified in the logged chat data. The patterns that emerged in each group are narratively described. Finally, a joint display (Multimedia Appendix 1) was created to show the expansion meta-inferences drawn from the merged quantitative and qualitative data.

### Ethical Considerations

The study was approved by the Swedish Ethical Review Authority (Dnr: 2019‐01632 and Dnr: 2020‐06882) and conducted according to the Declaration of Helsinki [42]. Informed written consent was obtained from the participants before the study. The participants were informed that their participation was voluntary, including consent to retrieve the logged chat data for research analysis. All the data were pseudonymized and kept confidential. No identifiable information was linked to the participants in the presentation of the results. The research data collected through the mobile app were securely stored on a backend server at Karolinska Institutet.

## Results

### Characteristics of Participants

Thirty-five FCs of people with dementia living at home completed the pre- and post-intervention evaluations. The FCs were aged 42-85 years (mean 69.4, SD 11.9 years) and most (28/35, 80%) were women and partners, including spouses and cohabitating partners (24/35, 68.6%), who lived together with the person with dementia (26/35, 74%). Almost 86% (30/35) of the people with dementia received respite care (data not shown). The baseline characteristics are summarized in Table 1.

**Table 1.** Baseline characteristics of the study sample (N=35).

Variable	Participants (N=35)
Age (years), mean (SD; range)	69.4 (11.9; 42-85)
Sex, n (%)	
Female	28 (80)
Male	7 (20)
Relationship to the person with dementia, n (%)	
Partner (spouse and cohabitating partner)	24 (68.6)
Others (ie, adult children)	11 (31.4)
Living arrangement of the person with dementia, n (%)	
Living with a family member	26 (74.3)
Living alone	9 (25.7)

### Caregivers’ Stress and Depressive Symptoms

The mean score of FCs’ caregiver stress was marginally higher postintervention (mean 24.1, SD 9.3) compared to preintervention (mean 23.9, SD 9.2), although this difference was not statistically significant. The mean score of FCs’ depressive symptoms declined between preintervention (mean 6.5, SD 5.1) and postintervention (mean 6.2, SD 5.2), but was also not found to be statistically significant.

#### Association Between Participation in the Intervention and Caregiver Stress

The generalized multivariable regression analyses (Table 2) showed that the intervention did not have a significant association between the participation in the intervention on caregiver stress (dependent variable) after adjusting for age, sex, and relationship to the person with dementia (β=0.171, α=.05; *P*=.86).

**Table 2.** Generalized multivariable association between family caregiver’s participation in the intervention and i) caregiver stress and ii) depressive symptoms, analyzed using a generalized linear model with a Gaussian distribution and log link function (number of observations: n=70; number of groups=35).

Characteristics	Caregiver stress		Depressive symptoms	
	β coefficient (95% CI)	*P* value	β coefficient (95% CI)	*P* value
Age (in years)	−0.133 (−0.471 to 0.205)	.44	−0.118 (−0.278 to 0.042)	.15
Sex: female (reference group: male)	−5.72 (−12.50 to 1.05)	.10	−2.44 (−5.364 to 0.75)	.13
Relationship to the person with dementia: partner (reference group: others)	5.06 (−3.56 to 13.68)	.25	5.28 (1.201 to 9.349)	.01
The intervention: postintervention (reference group: preintervention)	0.171 (−1.76 to 2.10)	.86	−0.293 (−2.057 to 1.471)	.75

#### Association Between Participation in the Intervention and Depressive Symptoms

The generalized multivariable regression analyses (Table 2) showed that the intervention did not have an association with depressive symptoms (dependent variable) after adjusting for age, sex, and relationship to the person with dementia (β=−0.293, *α*=.05; *P*=.75). However, a significant association was found between the relationship to the person with dementia and depressive symptoms, as partners, including spouses and cohabitating partnerFCs (β=5.28, α=.05; *P*=.01), were more likely to report higher levels of depressive symptoms than others (ie, adult children).

#### Type of Support in the Chat Log Data

The most common types of support that emerged between the FCs and the social care professionals in the logged chat data in terms of chat entries were informational (278/587, 47.4%) and emotional (192/587, 32.7%), followed by instrumental support (26/587, 4.4%). The other types of chat entries (91/587, 15.5%) mostly included logistics and information concerning the intervention (ie, confirming received messages and conveying start and end dates for the intervention).

##### Informational Support

Informational support was the most common type of support discussed by the FCs and the social care professionals in the chat. The social care professionals used the chat feature to guide the FCs to available offline and online information (ie, information about legal advice and dementia) and support services (ie, group support). Social care professionals also used the chat feature to direct the FCs to the web link feature of STAV and provide information regarding upcoming events (ie, lectures about yoga and mindfulness) and instrumental support (ie, home care and memory aid for the person with dementia). These messages went beyond daily greetings and check-ins, often consisting of probing questions to the FCs, such as asking them to name the diagnoses of the person with dementia or reflect on the symptoms and causes of their recent behaviors. The FCs often used the chat to update the social care professionals about the current state of the person with dementia that they cared for, which included physical and behavioral changes of the person with dementia, such as exhibiting signs of restlessness, wandering, withdrawal, apathy, and memory loss. The social care professionals and the FCs also used the chat feature to confirm new symptoms of the person with dementia with one another or to discuss conflicting accounts about the daily activities of the person with dementia at the daycare center who was experiencing memory loss.

The FCs often asked the social care professionals for coping strategies, such as keeping the person with dementia activated while waiting to return to respite care and setting boundaries with the person with dementia when they exhibited challenging behaviors (ie, suspicion, restlessness, repeating themselves, and anger). The social care professionals responded by educating the FCs on common symptoms of dementia and providing advice on how best to manage them, which included advising the FCs to change the topic of discussion or divert the conversation as a coping strategy. However, some complex conversations were challenging for the social care professionals and the FCs to discuss in the form of chat. In those cases, they reverted to other communication channels, such as the telephone or in-person meetings. Finally, as the intervention period was ending, most social care professionals made sure to provide the FCs with their contact information and navigated them to other support services, such as individual and group support for FCs provided by the municipality.

##### Emotional Support

Emotional support was the second most common type of support that emerged in the chat entries between the FCs and the social care professionals. The social care professionals provided support ranging from regular check-ins with the FCs, conveying their availability, or sending brief greetings, to more in-depth queries regarding the well-being of the FCs and the person with dementia they cared for. The chat feature provided the FCs an outlet to vent and share their negative emotions, such as feeling overwhelmed, frustration, fatigue, and guilt related to caregiving tasks (eg, medication responsibilities and coordination of health care services). The chat feature enabled the social care professionals to convey empathy and care to the FCs by sending messages that validated and reassured the caregivers regarding their negative emotions related to caregiving.

The social care professionals also used the chat to send self-care reminders, encouraging the FCs to prioritize their needs and accept support (ie, home care provided by the municipalities). The self-care reminders also focused on the FCs’ physical well-being, reminding them to breathe, rest, stay hydrated, and engage in stress-reducing activities. These prompts encouraged the FCs to reflect, and some caregivers responded that they met their self-care needs by engaging in activities such as spending time with loved ones (ie, children and grandchildren), baking, or exercising. Some social care professionals also encouraged the FCs to use the mindfulness and diary features of STAV for destressing and offloading purposes.

##### Instrumental Support

Instrumental support was the least common type of support found in the chat entries. Instrumental support mostly related to coordinating care, such as arranging walking aids to facilitate the person with dementia during transportation to and from medical appointments. The chat feature of STAV also enabled the FCs and the social care professionals to coordinate personal care (ie, foot care) for the person with dementia while attending respite care. The FCs’ chat conversations regarding instrumental support also included practical support, such as needing help to search for an item that the person with dementia had lost or misplaced at the senior daycare center. The chat feature enabled the social care professionals to accommodate and respond to the changing needs of the FC and the person with dementia. This could include rescheduling transportation or respite care of the person with dementia due to conflicting leisure activities (ie, vacation plans for the FCs and the person with dementia) and other respite activities of the person with dementia.

### Mixed Methods Analysis Results

#### Group 1: FCs With Decreased Caregiver Stress After Intervention (17/35, 48.6%)

FCs with decreased caregiver stress scores after the intervention generally had a higher number of chat message entries (mean 6.5) directed toward social care professionals, indicating regular engagement and active help-seeking behaviors. These FCs also received a relatively higher number of chat message responses (mean 10) from social care professionals, indicating timely and reciprocal engagement between the users to meet the FCs’ needs. Most FCs in this group with decreased caregiver stress also showed improvements (n=8) or no change (n=3) in depressive symptoms.

Through the chat message entries, the FCs vented and shared their negative experiences and emotions related to caregiving with the social care professional, such as feeling frustrated, guilty, and overwhelmed with caregiving tasks (eg, having responsibilities regarding medication administration, coordination of health care services, and respite care).

*That is just how I feel; guilt and duty, and a mother (the person with dementia) that is demanding in the relationship…*

[FC 1057]

*I feel like a hotel hostess with medication responsibilities*.

[FC 1040]

These FCs often sought and received tailor-made emotional, instrumental, and informational support, such as coping advice and information about relevant services to address caregiving challenges. Responses from social care professionals included giving relevant information by entering direct chat messages or providing relevant web links.

*From a psychological perspective, the disease progression can be divided into different phases (provides web link*).

[Social care professional 1010]

The social care professionals provided emotional support by validating the FCs’ emotions, offering empathy and reassurance, and encouraging the caregivers to adopt self-care behaviors and activities. One social care professional also directed the FCs to other support features of STAV (ie, the diary feature) for offloading purposes.

…*Remember to breathe and take care of yourself as well!*

[Social care professional 1001]

The chat message entries also focused on information and provision of instrumental support, including coordinating care for the person with dementia regarding aids (eg, memory aids for the person with dementia), personal care, respite care, and visits to health care services.

*Hi…I have arranged foot care for (the person with dementia) on Tuesday. Regards*.

[Social care professional 1011]

#### Group 2: FCs With No Changes in Caregiver Stress After Intervention (5/35, 14.3%)

For the FCs with no change in caregiver stress after the intervention, all except one provided (n=0‐2 messages) and received a low frequency of chat message entries (n=1‐5 messages). None of the chat messages addressed instrumental support, and only a few of the messages by the FCs conveyed the need for information about, for example, caregiver support and coping advice, as well as emotional support (ie, frustration related to home care services for the person with dementia).

*Hello, in contact with home health care, it is sometimes difficult as an FC to be understood. I feel frustrated that my concerns are not being heard or responded to*…

[FC 1042]

The messages from the social care professionals in this group generally consisted of conveying their availability or reminders for the FCs to stay in touch; some provided fixed time slots, while others adjusted their availability to meet the needs of the FCs. The chat entries by the social care professionals also consisted of generic holiday greetings or acknowledgments (eg, “That’s great!”) regarding updates concerning the person with dementia, which included daily activities and respite care. Thus, these messages were less individualized than the messages sent for the FCs in group 1 and often lacked deeper engagement in terms of reassurance and validation, which were conveyed less frequently.

*Welcome! Now you can reach out to me; I’m available here*.

[Social care professional 1009]

Although this group did not have any change in caregiver stress, all but one of the FCs showed improvements (n=3) or no change (n=1) in depressive symptoms.

#### Group 3: FCs With Increased Caregiver Stress After Intervention (13/35, 37.1%)

The FCs with an increase in caregiver stress postintervention generally had frequent engagements (mean 6) in the chat and expressed the need for emotional and informational support to a greater extent than the FCs in group 2 (no change in caregiver stress), which was conveyed by asking the social care professionals for coping advice on how to activate the person with dementia and on managing challenging behaviors of the person with dementia, such as, frustration and memory loss.

*Perhaps (I) can ask if there is anything I can activate (the person with dementia) with now when we do not have (access) the senior daycare centre*.

[FC 1051]

For this group, the social care professionals generally provided a higher number of chat message responses (mean 9). The social care professionals also conveyed their availability in the chat and supported the FCs by validating and checking in with them. The provided support was generally less generic than the support identified for group 2. For instance, one social care professional who worked in a senior daycare center updated an FC about the current emotional state of the person with dementia they cared for. However, the emotional support focused less on reassuring the FCs about their expressed emotions related to caregiving (ie, frustration) as identified in the support provided for group 1.

*Hi [FC], how nice that you respond [in the chat], yes of course it’s nice to get out without having to have a bad conscience and that he (the person with dementia) is not alone*.

[Social care professional 1008]

Furthermore, the social care professionals also guided the FCs to additional support services through the chat by sending information about upcoming lectures and relevant web links to educate the FCs about dementia. The chat was also used to inform the FCs about available support services and to direct them to social aid officers in the municipalities for additional support (eg, respite care for people with dementia) to relieve the FCs in their role as caregivers.

*Hi (FC) …call the social aid officer for information about potential services that can support your partner (the person with dementia) when you need to get away from the house to run your errands, (your) interests*.

[Social care professional 1007]

A few of the FCs in this group also needed direct instrumental support, including assistance to book or reschedule transport for the person with dementia to and from activities (ie, health care visits) or help with searching for misplaced items belonging to the person with dementia at the senior daycare center.

*Hi, mum (the person with dementia) has misplaced her house keys. Could you look for them at [the senior day care centre]?*

[FC 1055]

While the FCs in this group showed an increase in caregiver stress postintervention, most of the FCs showed improvements (n=5) or no change (n=3) in depressive symptoms. However, no differences were found in the chat entries in terms of support needs expressed by the FCs and provided by the social care professionals between the FCs who showed improvements or no changes and those who showed a deterioration in depressive symptoms.

## Discussion

### Principal Findings

Participation in the intervention did not have a significant association with caregiver stress and depressive symptoms among the FCs of people with dementia in this study. However, the effectiveness of internet-based or mobile app interventions for FCs of older adults has previously been shown for psychological outcomes, including reduction in depression and caregiver stress [43]. The results showed that informational support, followed by emotional support, was the most mentioned support type. A review [44] reports that some RCTs based on multicomponent interventions, including the provision of informational support, have shown positive impacts on outcomes such as perceived stress and depression. However, to be considered useful, the information provided must be tailor-made to the needs of the FCs [44]. This impact was potentially indicated among the subgroup of FCs with decreased stress who received support in the chat by the social care professionals, tailored to their needs (ie, reassurance, validation, and information) to a greater extent than those FCs with no change or increase in caregiver stress postintervention. However, it is important to note that the impact of informational support is difficult to assess since it is often integrated as a component of a broader intervention [44], as for the mobile app STAV, where web links to available resources are included. Although these results are not generalizable, the mixed methods analysis gave important insights into how the changes in the primary outcome, that is, caregivers’ stress, were related to specific types of support provided by the social care professionals through chat conversations with FCs.

The heterogeneity of FCs of people with dementia also needs consideration when designing online-based interventions. One of the main implementation challenges includes the condition of the care recipient (ie, the variation in the severity, type, and stage of dementia) [45], which was not considered in this study. However, the FCs with no change in stress between pre- and post-intervention generally had low engagement in the chat, which might be indicative of a low support need related to caring for a family member with early-stage dementia, as reported in a previous qualitative evaluation of the intervention [46]. Another important factor that needs consideration is the relationship between the FCs and the person with dementia. High caregiver stress is more prevalent among FCs who cohabitate with the person with dementia than among noncohabitating FCs [47]. In this study, a significant association was found, as partners, including spouses and cohabitating partners, were more likely to report higher levels of depressive symptoms than others (ie, adult children). These results are in line with a recent Swedish study where spouses reported significantly higher mean scores in negative outcomes of informal caregiving, including “physical and psychological stress” and “trouble finding time to spend with friends,” than nonspouse FCs [7].

The interaction between the FCs and the social care professionals through the chat feature of STAV could also have impacted the outcome of the intervention on caregiver stress and depressive symptoms. A systematic review [44] reports that interacting directly with professionals (ie, nurses, social workers, or occupational therapists) through internet-based interventions provides FCs of people with dementia living at home with a positive experience. Furthermore, receiving tailor-made emotional and practical advice can decrease feelings of isolation for FCs [44]. Research [45] further suggests that higher engagement and lower intervention dropout rates occur when human contact (eg, health care professionals, research teams, and coaches) is incorporated in the intervention, as opposed to an intervention with no human contact, depending on external sources of interaction (ie, providing a social media page or a telephone number) [45]. Nurses described in a Norwegian study the importance of offering FCs of people with dementia the opportunity to vent their frustrations remotely through telecare using a web camera and a web forum [48]. Similarly, the intent of the chat feature of STAV in this study was to provide support remotely. The results potentially indicate that receiving individualized emotional support in terms of validation and self-care prompts, as opposed to generic validation, had a greater impact on caregiver stress. This was identified for the group of FCs with decreased caregiver stress postintervention, who also mostly showed improvement in depressive symptoms.

There were varied levels of engagement between the FCs and the social care professional through the chat. The FCs with decreased and increased stress had relatively frequent interaction, while FCs with no change generally had low engagement. A previous qualitative evaluation of the intervention [46] reported that some of the FCs and the social care professionals were unfamiliar with each other before partaking in the intervention. Some of the FCs also received supplementary forms of caregiver support during the intervention period (ie, face-to-face support groups). This variance between the interplay of interacting through the chat and meeting in person needs consideration, since text-based platforms, including the mobile app STAV, lack nonverbal cues, which can be expressed through in-person conversations (ie, touch and facial expressions) [49]. This could have contributed to the engagement levels of the FCs and the social care professionals.

Recent research has shown that digital literacy skills and education needs of FCs of people with dementia are significantly associated with the use of mHealth apps [50]. The fact that most of the FCs in this study were older partners, including spouses and cohabitating partners, of people with dementia could have contributed to some challenges with digital literacy. The motivation, privacy concerns, training, and education of the FC of people with dementia further determine eHealth technology implementation [51]. Therefore, it is suggested [51] to support FCs in the use of technology before implementation. It is also important to provide the FCs with adequate time for practice and provide in-person assistance in case of technical issues [51]. Most of the FCs received a brief introduction to the mobile app STAV used in this intervention. Members of the research team also assisted with technical issues when needed. However, this was mostly conducted via telephone conversations due to the COVID-19 pandemic and at the wish of the FCs. Thus, conducting in-person training sessions with the FCs could have resulted in increased uptake of the intervention for the FCs who had challenges with digital literacy. The duration of the intervention, consisting of 8 weeks, could also have impacted the intervention outcomes because a lack of digital literacy can increase the need for additional effort and time to adapt to the new technology. Subsequently, some of the FCs in this study might have needed an onboarding process where enough time was provided to familiarize themselves with the new technology at their own pace [52]. Finally, other factors that could have affected the associations of the intervention were not considered, such as the education and financial level of the FCs and whether the caregivers lived with or had support from other family members.

### Limitations and Future Research

The participants of this study consisted mostly of women. Even though the sex distribution is representative of FCs of people with dementia in Sweden [6], a larger proportion of male FCs is needed to explain sex differences in the association between the intervention and the outcome measures. The 8-week duration of the intervention might be too short to bring about significant changes in caregiver stress and depressive symptoms. Digital interventions such as STAV have the potential to reduce selection bias by reaching individuals who are typically underrepresented, such as those living in remote settings [53]. It is possible that the study primarily attracted FCs who were motivated to receive the intervention and were digitally literate. As a result, FCs who needed support but found it challenging to use a mobile app as a mode of communication could have been excluded, potentially leading to a selection bias among FCs of people with dementia. Therefore, the use of the mobile app should serve as a complement, rather than a replacement for traditional means of support. Future studies should also consider the heterogeneity of FCs to the person with dementia, such as the stage of dementia of the care recipient. Future studies could integrate outcome measures with logged analytics data to further examine associations between the digital intervention and the outcome measures.

### Conclusions

Patterns observed in the mixed methods analysis at the subgroup level suggest that tailored support provided through asynchronous and synchronous chat delivered through a mobile app may be associated with a potential decline in caregiver stress among FCs of people with dementia living at home. However, a full-scale study with a larger sample size is necessary to confirm these findings and generalize the results of this pilot study to a broader population. These findings highlight the importance of providing frequent and tailor-made informational, emotional, and instrumental support to meet the individual needs of FCs of people with dementia. The findings from this study can help community-based social care providers plan and organize the content of digital support provided for FCs of people with dementia living at home.

## Supplementary material

10.2196/75113 Multimedia Appendix 1 Joint display: integration of quantitative and qualitative results.

10.2196/75113 Checklist 1 GRAMMS checklist.

## Abbreviations

*DSM-IV*: *Diagnostic and Statistical Manual of Mental Disorders, Fourth Edition*
FC: family caregiver
GRAMMS: Good Reporting of a Mixed Methods Study
mHealth: mobile health
PHQ-9: Patient Health Questionnaire
STAV: STöd till AnhörigVårdare
WHO: World Health Organization
ZBI-12: 12-item Zarit Burden Interview
