# Supplementary material for: Professional Support Through a Tailor-Made Mobile App to Reduce Stress and Depressive Symptoms Among Family Caregivers of People With Dementia: Mixed Methods Pilot Study
Source: JMIR Form Res. 2025 Sep 30;9:e75113. doi: 10.2196/75113 (PMC12483335; doi:10.2196/75113)
Supplement: Multimedia Appendix 1 [file formative-v9-e75113-s001.pdf]

Quantitative	Qualitative				Mixed
Change in caregiver stress mean scores (pre-post intervention)	No. chat entries	Instrumental	Emotional	Informational	Meta-inferences
**Group 1:** FCs with decreased change scores (n=17)	Entries by FCs: 111 (mean=6.5, range 0-22)<br><br>Entries by SCPs: 166 (mean=10, range 0-21)	Hi… I have arranged foot care for [the PWD] on Tuesday. Regards. [Social care professional 1011]	I feel like a hotel hostess with medication responsibilities. [FC 1040]	From a psychological perspective the disease progression can be divided into different phases [provides weblink]. [Social care professional 1010]	The FCs generally had frequent engagement in the chat indicating active help-seeking behaviours. These FCs often sought and received tailor-made emotional instrumental and informational support. The emotional support related to e.g., venting about feelings of guilt and frustration. The SCPs validated the FCs' emotions, offering, reassurance and encouraging the caregivers to adopt self-care behaviours and activities. Here the SCPs also provided information about available instrumental support (i.e., respite care) and coordinated direct instrumental support (i.e., personal care for the PWD). Most of the FCs in this group showed improvements or no change in depressive symptoms.
**Group 2:** FCs with no change scores (n=5)	Entries by FCs: 39 (mean=8, range 0-34)<br><br>Entries by SCPs: 27 (mean=5, range 1-19)	No data available	Hello, in contact with home health care, it is sometimes difficult as an FC to be understood. I feel frustrated that my concerns are not being heard or responded to… [FC1042]	Hi, now in the heat wave, we [at the senior day care centre] just go for [a] short walk, sit in the shade and enjoy instead. Best regards [Social care professional 1011]	All except one of the FCs generally had low engagement with the SCPs in the chat. The messages were related to emotional support (i.e., venting frustrations related to caregiving) and informational support (i.e., coping advice). The messages from the social care professionals were more generic and less individualised in the form of acknowledgements and daily updates concerning the PWD. All but one of the FCs in this group showed improvements or no change in depressive symptoms.
**Group 3:** FCs with increased scores (n=13)	Entries by FCs: 80 (mean=6, range 0-20)<br><br>Entries by SCPs: 112 (mean=9, range 0-28)	Hi, mum [the PWD] has misplaced her house keys. Could you look for them at [the senior day care centre]? [FC 1055]	Hi [FC], how nice that you respond [in the chat] 😊, yes of course it's nice to get out without having to have a bad conscience and that he [the PWD] is not alone [Social care professional 1008]	Perhaps can ask if there is anything I can activate [the PWD] with now when we do not have [access to] the senior daycare centre. She [The PWD] has been promised to come back at the end of August in any case … [FC1051]	The FCs generally had frequent engagement in the chat. Chat messages related to all three types of support. Informational support included coping advice and navigation to upcoming lectures and available support services. The emotional support provided by the SCPs was generally less generic than for Group 2, the messages were less focused on reassuring the FCs than seen for Group 1. The instrumental support was mostly related to coordinating logistics i.e., booking or rescheduling transport for the PWD. Most of the FCs in this group showed improvements or no change in depressive symptoms.

Legend: FC(s): Family caregivers, SCP(s): Social care professionals, PWD: Persons with dementia.
