# Supplementary material for: Professional Support Through a Tailor-Made Mobile App to Reduce Stress and Depressive Symptoms Among Family Caregivers of People With Dementia: Mixed Methods Pilot Study
Source: JMIR Form Res. 2025 Sep 30;9:e75113. doi: 10.2196/75113 (PMC12483335; doi:10.2196/75113)
Supplement: Checklist 1 [file formative-v9-e75113-s002.docx]

Good Reporting of a Mixed Methods Study (GRAMMS) checklist.

| Guideline | Section: page |
| --- | --- |
| Describe the justification for using a mixed methods approach to the research question | Design: page 5 |
| Describe the design in terms of the purpose, priority and sequence of methods | Design: page 5 |
| Describe each method in terms of sampling, data collection and analysis | Setting and participants:  page 5  Data collection: page 6-7  Data analysis: page 7-8 |
| Describe where integration has occurred, how it has occurred and who has participated in it | Design: page 5  Results: Page 8-15 |
| Describe any limitation of one method associated with the present of the other method | Discussion: page 15-17  Limitations and future research: page 17-18 |
| Describe any insights gained from mixing or integrating methods | Discussion: page 15-17  Limitations and future research: page 17-18  Conclusion: page 18 |

*O'Cathain A, Murphy E, Nicholl J. The quality of mixed methods studies in health services research. J Health Serv Res Policy. 2008;13(2):92-98.*
